# ‘Setting the Benchmark’ Part 3: Contextualising the Match Demands of Specialised Positions at the FIFA Women’s World Cup Australia and New Zealand 2023

**DOI:** 10.5114/biolsport.2025.139857

**Published:** 2024-06-04

**Authors:** Paul S. Bradley

**Affiliations:** FIFA, Zürich, Switzerland

**Keywords:** Match Analysis, Soccer, Female, Position, High Intensity, International, Evolution

## Abstract

This study aimed to benchmark the match demands of specialised positions at the FIFA Women’s World Cup Australia and New Zealand 2023. With FIFA’s official approval, all sixty-four games at the tournament were analysed using an optical tracking system alongside FIFA’s Enhanced Football Intelligence metrics. During a typical match, defensive and central midfielders covered 5–15% more total distance than centre backs, wide defenders and centre forwards (P < 0.01; Effect Size [ES]: 0.6–1.8). The distances covered at higher intensities (≥19.0 and ≥23.0 km · h^-1^) were 18–89% and 88–163% greater in attacking midfielders, wide midfielders, wide forwards and centre forwards than other positions (P < 0.01; ES: 0.5–2.0 and ES: 1.0–1.3, respectively). Regarding offers made to receive the ball, defensive and central midfielders, attacking midfielders and centre forwards moved more between the lines than centre backs, wide defenders and wide midfielders (P < 0.01; ES: 1.0–1.9). Movements in behind lines were more common for offensive roles such as attacking midfielders, wide midfielders, wide forwards and centre forwards than other positions (P < 0.01; ES: 0.9–2.3). Regarding pressing events, direct pressure was highest for defensive and central midfielders compared to other positions (P < 0.05; ES: 0.5–1.3) and indirect pressure was greater for central midfielders, attacking midfielders, wide midfielders and centre forwards compared to centre backs and wide defenders (P < 0.01; ES: 0.9–2.3). A basic within tournament positional comparison revealed that centre backs and centre forwards demonstrated pronounced changes in their relative sprint distances from Canada 2015, France 2019 through to Australia and New Zealand 2023. These findings could be valuable to benchmark the contemporary positional demands of women’s international football, while also providing a framework to design role-specific training drills.

## INTRODUCTION

An examination of the match-play characteristics of women’s football demonstrates that the technical and tactical performances of teams have steadily progressed over time [1]. However, one area of performance that has evolved exponentially in the women’s game over the last decade is the speed and the intensity of match-play. For instance, high-intensity running and sprinting distance on a team level has increased by around 20–30% between the FIFA Women’s World Cup Canada 2015 and France 2019 [2]. Accordingly, given such accelerated rates of physical development, it is crucial that the match demands of contemporary competitions such as the FIFA Women’s World Cup Australia and New Zealand 2023 are analysed and documented. Findings from such analyses could be valuable to benchmark the current intensity of international female match-play, while also providing a framework for the development of female-specific drills via the replication of such demands [3–5]. Besides, there is also an onus on practitioners to be cognisant of modern-day demands, as an intensification of match-play could be one of a multitude of risk factors associated with increased injury prevalence in players [6].

It is customary for studies exploring the match demands of elite female players to find that performances are highly dependent upon the positional role in the team [2, 4, 7–10]. Some of these studies employed broad positional categories such as defenders, midfielders and forwards, while others assigned up to four or five different positions. Recent findings demonstrated that using eight to eleven outfield roles to quantify the match demands of elite male players resulted in highly distinguishable movement characteristics compared to broad categories [11, 12]. However, research to date has yet to partition international female players into highly distinctive outfield positions. Redressing this shortcoming could be highly relevant to the women’s football community, especially if the data was from a recent tournament such as the FIFA Women’s World Cup 2023. Thus, a study that breaks female international players down into specialised outfield positions and even individual player roles may enable practitioners to use match physical performance trends as a blueprint to create position- and individual-specific drills [13]. Researchers have created position-specific drills that tax the relevant physical attributes of elite male players, while simultaneously mimicking some of the most commonly occurring technical and tactical actions [14]. Thus, a detailed match analysis of the FIFA Women’s World Cup 2023 may facilitate this process for women’s football. In addition, it is unclear which positions in modern female international match-play contribute more physically in- or out-of-possession of the ball or how their work-rates are distributed across halves. Gaining a deeper understanding of this latter point could be advantageous given new directives have increased game time since the FIFA Women’s World Cup France 2019.

To offer practitioners more nuanced and actionable positional insights, match physical analyses should ideally be layered with context [15–17]. As a reductionist approach has typically been applied to women’s match demands research [2, 4, 7–10, 18], further investigations are warranted that use an array of tactical and technical metrics alongside match physical performance measures. As FIFA tournaments now use Enhanced Football Intelligence metrics [11, 19, 20], this may progress our knowledge of such a complex sport. Moreover, limited research exists that has revealed whole tournament positional trends in conjunction with pertinent examples of individuals in selected positions. Using such a dual approach may further our understanding of the dynamic interplay between physical, technical and tactical factors [11, 19]. Thus, this study aimed to benchmark the match demands of specialised positions at the FIFA World Cup Australia and New Zealand 2023.

## MATERIALS AND METHODS

### Sample

With FIFA’s official approval, all sixty-four games at the FIFA Women’s World Cup Australia and New Zealand 2023 were analysed. The data provider assigned eight outfield roles to enable positional differences to be determined. Games were filtered so only players who completed the entire match were evaluated. This fell in line with the physical analyses conducted for the previous two editions of the FIFA Women’s World Cup (Canada 2015 and France 2019), in addition to the FIFA World Cup Qatar 2022 [2, 11, 19]. This enabled 806 player observations to be analysed across various positions (210 centre backs [CB], 207 wide defenders [WD], 60 defensive midfielders [DM], 118 central midfielders [CM], 38 attacking midfielders [AM], 63 wide midfielders [WM], 34 wide forwards [WF], and 76 centre forwards [CF]). As this data were freely available, no ethical approval was required [21].

### Match Tracking System

FIFA Women’s World Cup 2023 games were analysed using an optical tracking system that operated at 25 Hz (TRACAB, Chyron Hego, Sweden). This systems validity has been quantified to verify the capture process and subsequent accuracy of the data [22]. After system calibration (e.g., pitch locations of known distances) and various quality control processes (e.g., regularly checking the tracking of players during games), the data captured were analysed using match analysis software. This produced a data set on each player’s activity pattern during a match using female-specific speed zones [2].

### Speed Zones

Players’ activities were coded into the following:

– Zone 1 (0.0–6.9 km · h^-1^)
– Zone 2 (≥7.0–12.9 km · h^-1^)
– Zone 3 (≥13.0–18.9 km · h^-1^)
– Zone 4 (≥19.0–22.9 km · h^-1^)
– Zone 5 (≥23.0 km · h^-1^)

Analyses primarily reported physical metrics that provided insights into the volume (total distance) and intensity of match-play (high-intensity and sprinting distance). Total distance represented the sum of the ground covered in all speed zones. High-intensity distance consisted of the aggregation of zones 4 and 5 (≥19.0 km · h^-1^), while sprinting exclusively included zone 5 distance (≥23.0 km · h^-1^). Additionally, these metrics were analysed based on possession status or if the ball was out of play. Top speeds attained in games were also quantified across all positions.

### FIFA’s Enhanced Football Intelligence Metrics

To further contextualise the physical data, FIFA’s Enhanced Football Intelligence metrics were also quantified. This included the coding of events such as the number of passes, successful passes, crosses, ball progressions, total offers made to receive and various movement types, in addition to applied pressures. Event definitions can be found in freely available documentation [20, 23].

### A Within Tournament Sprinting Analysis Across Three FIFA Women’s World Cups

The speed zones adopted at the FIFA Women’s World Cup 2023 were identical to those at the Canada 2015 and France 2019 editions, but the latter two utilised a different optical tracking system (STATS LLC, USA). Thus, it would be challenging to compare positions between tournaments. Consequently, a within tournament analysis of selected positions at the high and low end of the sprinting continuum was conducted instead. As the evolution of match physical performances are more pronounced at zone 5 [2], only sprinting distances were evaluated in this way. To further aid this comparison, only relative measures were calculated (e.g., zone 5 distance as a percentage of total distance). Moreover, some positions were combined and/or omitted from the 2023 data as previous analyses did not categorise players into eight outfield roles. This allowed profiling of 2154 player observations across the 2015, 2019 and 2023 tournaments, respectively (161, 199, 210 centre backs [CB], 151, 175, 207 wide defenders [WD], 160, 191, 178 defensive/central midfielders [DM/CM], 98, 98, 97 wide midfielders/forwards [WM/WF], and 73, 80, 76 centre forwards [CF]).

### Statistical Analyses

Analyses were conducted using statistical software (SPSS Inc, Version 26.0, IBM Corp, USA). Descriptive statistics were calculated on each variable. To verify normality, z-scores were obtained through dividing the skewness and kurtosis values by their standard error. Differences across role, possession status, halve and tournament were determined using factorial analysis of variance (ANOVA). In the event of a significant difference occurring, univariate analyses using Bonferroni-corrected pairwise comparisons were employed. Statistical significance was set at *P* < 0.05. Quadrant plot analysis composed of a simple percentage distribution computation [11, 19]. The coefficient of variation (CV) was used to determine the data spread across metrics. Effect sizes (ES) were computed to determine the meaningfulness of any differences. The ES magnitudes were classed as trivial (< 0.2), small (> 0.2–0.6), moderate (> 0.6–1.2) and large (> 1.2). Pearson’s coefficients were used for correlation analyses and the magnitudes of the associations were regarded as trivial (*r* < 0.1), small (*r* > 0.1–0.3), moderate (*r* > 0.3–0.5), large (*r* > 0.5–0.7), very large (*r* > 0.7–0.9), and nearly perfect (*r* > 0.9). Values are presented as means and standard deviations unless otherwise stated.

## RESULTS

### Benchmarking & Variation

Figures 1A-D benchmark and display the variation across each position from a physical perspective. Examples at both ends of the physical continuum are also highlighted to add context and aid interpretability. Figure 1A indicates that CM and DM covered 5-15% more total distance than CB, WD and CF (P<0.01; ES: 0.6-1.8). Moreover, AM and WF have the highest coefficient of variation for the total distance covered (CV: 9.0-9.8%) compared to CB, WD, DM, CM and CF (CV: 6.4-8.0%). Figure 1B reveals that the distances covered at high-intensity (≥19.0 km · h^-1^) were 18–89% greater in AM, WM, WF and CF compared to CB, WD, DM and CM (*P* < 0.01; ES: 0.5–2.0). While the distances covered sprinting (≥23.0 km · h^-1^) were 88–163% higher in WD, AM, WM, WF and CF compared to CB, DM and CM (*P* < 0.01; ES: 1.0–1.3; Figure 1C). Moreover, CM and CF exhibited the greatest coefficient of variation (CV: 37.7–37.9% and 66.4–73.9%) for the distances covered at higher intensities (≥19.0 and ≥23.0 km·h^-1^, respectively) compared to other positions (CV: 21.8–34.6% and 37.7–57.6%). Figure 1D revealed that the top speeds attained during games were higher for AM, WM, WF and CF compared to DM and CM (*P* < 0.05; ES: 0.5–0.7). These top speed efforts varied across position (CB: 6.5%, WD: 6.2%, DM: 7.1%, CM: 6.6%, AM: 6.8%, WM: 5.8%, WF: 6.9%, CF: 6.9%). The top ten speeds indicated that offensive positions such as CF (30%), WF (20%) and AM (20%) accounted for 70% of these efforts.

**FIG. 1A:**
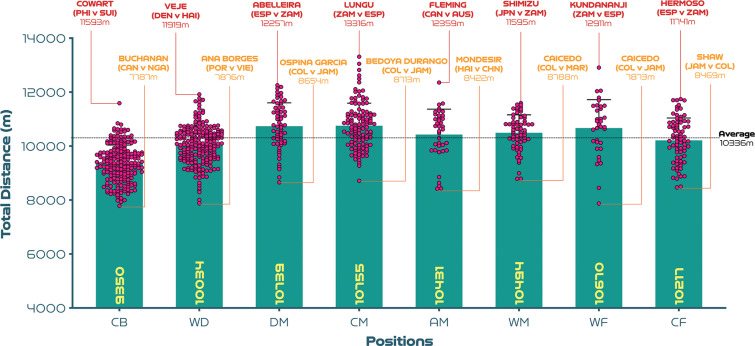
Total distance: average per game and variation for each positional role at the FIFA Women’s World Cup 2023. Data normalised for 90+ minutes (excludes goalkeepers and extra time). CB = centre back, WD = wide defender, DM = defensive midfielder, CM = central midfielder, AM = attacking midfielder, WM = wide midfielder, WF = wide forward, CF = centre forward. Red = highest value per position, orange = lowest value per position. Examples highlight both the individual player and the specific match.

**FIG. 1B:**
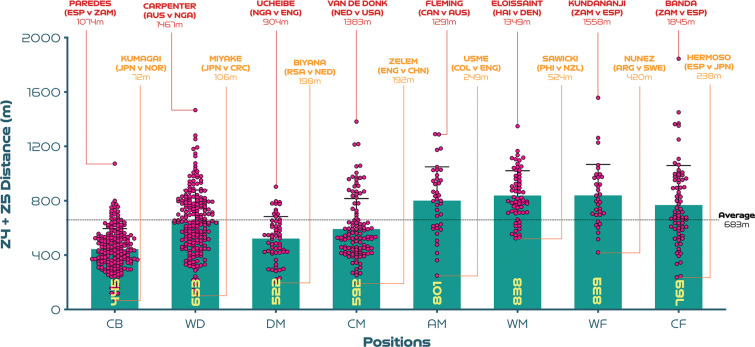
High-intensity distance (Zones 4 + 5 [Z4 + Z5], ≥19.0 km · h^-1^): average per game and variation for each positional role at the FIFA Women’s World Cup 2023. Data normalised for 90+ minutes (excludes goalkeepers and extra time). CB = centre back, WD = wide defender, DM = defensive midfielder, CM = central midfielder, AM = attacking midfielder, WM = wide midfielder, WF = wide forward, CF = centre forward. Red = highest value per position, orange = lowest value per position. Examples highlight both the individual player and the specific match.

**FIG. 1C:**
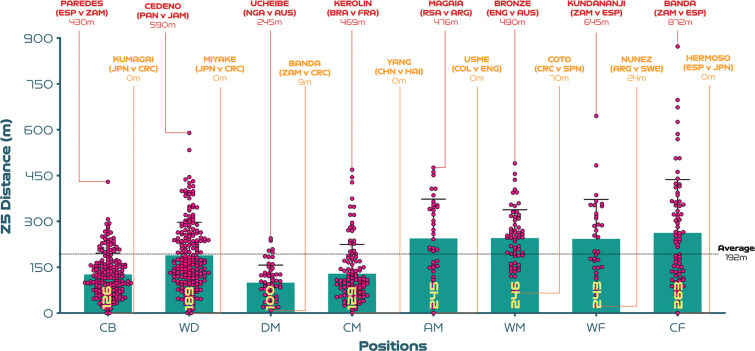
Sprint distance (Zone 5 [Z5], ≥23.0 km · h^-1^): average per game and variation for each positional role at the FIFA Women’s World Cup 2023. Data normalised for 90+ minutes (excludes goalkeepers and extra time). CB = centre back, WD = wide defender, DM = defensive midfielder, CM = central midfielder, AM = attacking midfielder, WM = wide midfielder, WF = wide forward, CF = centre forward. Red = highest value per position, orange = lowest value per position. Examples highlight both the individual player and the specific match.

**FIG. 1D:**
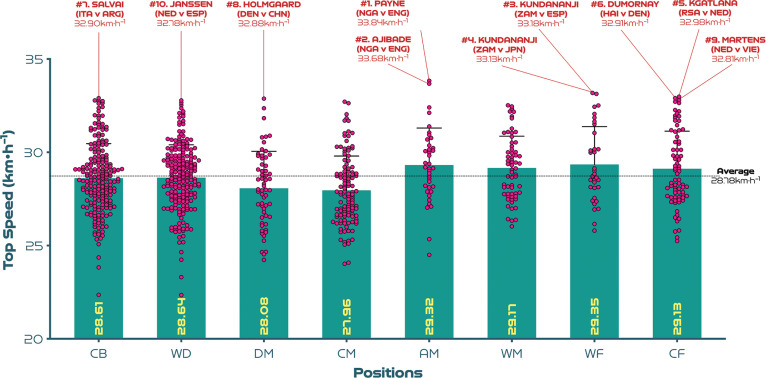
Top speeds: average per game and variation (including top ten speeds) for each positional role at the FIFA Women’s World Cup 2023. Data normalised for 90+ minutes (excludes goalkeepers and extra time). CB = centre back, WD = wide defender, DM = defensive midfielder, CM = central midfielder, AM = attacking midfielder, WM = wide midfielder, WF = wide forward, CF = centre forward. Red = top ten sprint speeds in the tournament. Examples highlight both the individual player and the specific match.

### Quadrant Plot Analysis

**FIG. 2:**
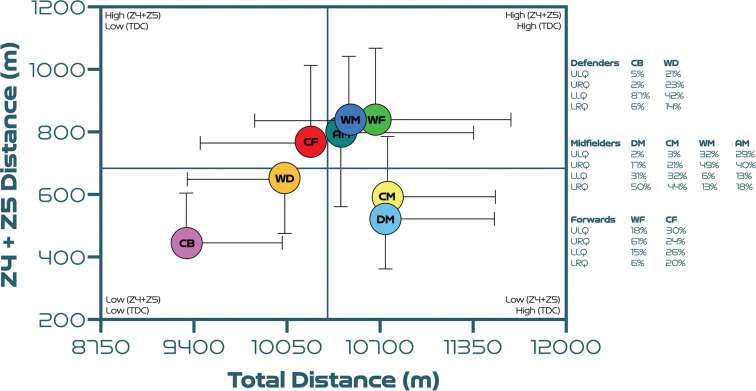
Total distance covered (TDC) versus high-intensity distance (Zones 4 + 5 [Z4 + Z5], ≥19.0 km · h^-1^) per positional role at the FIFA Women’s World Cup 2023. Data normalised for 90+ minutes (excludes goalkeepers and extra time). Crosshairs are based on the average for all roles. Circles denote the average per position and vertical and horizontal bars symbolise the standard deviation. CB = centre back, WD = wide defender, DM = defensive midfielder, CM = central midfielder, AM = attacking midfielder, WM = wide midfielder, WF = wide forward, CF = centre forward. Data on the right highlights the percentages of player observations in the various quadrants: ULQ = upper-left quadrant, URQ = upper-right quadrant, LLQ = lower-left quadrant, LRQ = lower-right quadrant.

Figure 2 uses quadrants to compare each position against one another. Regarding defenders, both CB and WD demonstrated moderate magnitude associations between total and high-intensity game distances (*r* = 0.37 and 0.36; *P* < 0.01). CB primarily occupied the lower-left quadrant (lower-left [LLQ] = 87%, lower-right [LRQ] = 6%, upper-left [ULQ] = 5% and upper-right quadrant [URQ] = 2%). Although WD occupied the lower-left quadrant most, some were also found in the upper-left and upper-right quadrants (LLQ = 42%, LRQ = 14%, ULQ = 21% and URQ = 23%). Regarding midfielders, a moderate magnitude correlation was found between a DM total and high-intensity game distances (*r* = 0.44; *P* < 0.01). DM were primarily found in the lower-right quadrant (LLQ = 31%, LRQ = 50%, ULQ = 2% and URQ = 17%). While only a small magnitude association was evident between a CM total and high-intensity game distances (*r* = 0.23; *P* < 0.05). Although CM occupied the lower-right quadrant more, some also deviated into the lower-left and upper-right quadrants (LLQ = 32%, LRQ = 44%, ULQ = 3% and URQ = 21%). Both WM and AM demonstrated small magnitude associations between the total and high-intensity game distances (*r* = 0.14 and 0.22; *P* > 0.05). WM mainly occupied the upper-right quadrant (LLQ = 6%, LRQ = 13%, ULQ = 32% and URQ = 49%). Similarly, AM were also upper-right quadrant dominant but more distribution occurred across other quadrants (LLQ = 13%, LRQ = 18%, ULQ = 29% and URQ = 40%). Regarding attackers, WF demonstrated a large magnitude association between total and high-intensity game distances (*r* = 0.51; *P* < 0.01). WF were mainly distributed in the upper-right quadrant (LLQ = 15%, LRQ = 6%, ULQ = 18% and URQ = 61%). In contrast, a small magnitude correlation was evident between a CF total and high-intensity game distances (*r* = 0.12; *P* > 0.05). CF were the position that fluctuated the most with no clear majority quadrant (LLQ=26%, LRQ=20%, ULQ=30% and URQ=24%).

### FIFA’s Enhanced Football Intelligence Metrics

Table 1 presents match events for each position at the FIFA Women’s World Cup 2023. Regarding distribution events, CB, WD, DM and CM passed more than AM, WF and CF (*P* < 0.01; ES:1.0–1.6). More crosses were performed by WD, AM, WM and WF than CB and DM (*P* < 0.01; ES: 0.8–1.4), while ball progressions were greater for AM, WM, WF and CF than CB, WD and DM (*P* < 0.05; ES: 0.5–1.2). Total offers made to receive the ball were greater for DM, CM, AM, WM, WF, CF than CB and WD (*P* < 0.01; ES: 0.7–1.7). Regarding offer movement type, DM, CM, AM and CF moved more between the lines than CB, WD and WM (*P* < 0.01; ES: 1.0–1.9). While DM and CM moved more in front of the lines than other positions (*P* < 0.01; ES:1.0–1.6). Movements in behind lines were more common for offensive roles such as AM, WM, WF and CF as opposed to defensive roles like CB, WD, DM and CM (*P* < 0.01; ES: 0.9–2.3). Total pressure applied was greater for all other positions versus CB and WD (*P* < 0.01; ES: 1.0–2.0). Direct pressure was highest for DM and CM compared to other positions (*P* < 0.05; ES: 0.5–1.3) and indirect pressure was greater for CM, AM, WM, WF and CF than CB and WD (*P* < 0.01; ES: 0.9–2.3).

**TABLE 1:** Match events for each positional role at the FIFA Women’s World Cup 2023.

Variable (No)	CB (*n* = 210)	WD (*n* = 207)	DM (*n* = 60)	CM (*n* = 118)	AM (*n* = 38)	WM (*n* = 63)	WF (*n* = 34)	CF (*n* = 76)	Basic Summary of Statistical Significance (*P*-values)
*Distribution Events*									

Passes	47 ± 29	52 ± 22	42 ± 17	41 ± 16	28 ± 10	35 ± 17	27 ± 13	23 ± 12	CB, WD, DM, CM > AM^b^, WF^b^, CF^b^
Passes Completed	40 ± 28	40 ± 22	33 ± 16	33 ± 15	20 ± 9	26 ± 15	20 ± 12	17 ± 11	CB, WD > AM^b^, WM^b^, WF^b^, CF^b^
Crosses	0 ± 0	3 ± 3	1 ± 2	2 ± 2	3 ± 3	3 ± 3	3 ± 3	2 ± 2	WD, AM, WM, WF > CB^b^, DM^b^
Ball Progression	1 ± 2	2 ± 2	1 ± 2	2 ± 2	3 ± 3	3 ± 2	4 ± 3	3 ± 2	AM, WM, WF, CF > CB^b^, WD^a^, DM^b^

*Total Offers Made & Offer Movement Type Events*									

Total Offers Made	13 ± 14	24 ± 19	41 ± 31	45 ± 27	43 ± 21	37 ± 18	39 ± 19	45 ± 26	DM, CM, AM, WM, WF, CF > CB^b^, WD^b^

Movement Offer In-Between	0 ± 1	1 ± 2	13 ± 12	15 ± 11	12 ± 11	4 ± 4	8 ± 8	14 ± 11	DM, CM, AM, CF > CB^b^, WD^b^, WM^b^

Movement Offer In-Front	2 ± 2	8 ± 7	22 ± 20	18 ± 14	6 ± 6	7 ± 6	6 ± 6	4 ± 7	DM, CM > CB^b^ WD^b^, AM^b^, WM^b^, WF^b^, CF^b^

Movement Offer In-Behind	0 ± 1	3 ± 4	1 ± 2	5 ± 7	12 ± 8	11 ± 7	13 ± 8	19 ± 14	AM, WM, WF, CF > CB^b^, WD^b^, DM^b^, CM^b^

Movement Offer Out-To-In	0 ± 0	0 ± 1	1 ± 1	1 ± 2	2 ± 2	1 ± 2	2 ± 2	1 ± 1	AM, WF > CB^b^, WD^b^, DM^b^, CM^b^, WM^a^, CF^b^

Movement Offer In-To-Out	0 ± 0	0 ± 1	1 ± 2	2 ± 3	3 ± 2	2 ± 2	3 ± 3	3 ± 3	CM, AM, WM, WF, CF > CB^b^, WD^b^

No Movement Offer	11 ± 12	12 ± 10	3 ± 3	4 ± 4	8 ± 7	12 ± 9	7 ± 6	4 ± 5	CB, WD, WM > DM^b^, CM^b^, CF^b^

*Pressing Events*									
Total Pressure Applied	8 ± 5	16 ± 8	25 ± 10	28 ± 14	30 ± 15	26 ± 14	33 ± 18	28 ± 13	DM, CM, AM, WM, WF, CF > CB^b^, WD^b^; WF > DM^b^

Direct Pressure	4 ± 3	6 ± 3	9 ± 5	8 ± 5	6 ± 4	6 ± 4	6 ± 4	4 ± 2	DM, CM > CB^b^, WD^b^, WM^a^, AM^a^, WF^a^, CF^b^

Indirect Pressure	4 ± 4	10 ± 6	16 ± 8	20 ± 12	24 ± 14	20 ± 9	27 ± 16	24 ± 13	CM, AM, WM, WF, CF > CB^b^, WD^b^; WF, CF > DM^a^, CM^a^

CB = centre back, WD = wide defender, DM = defensive midfielder, CM = central midfielder, AM = attacking midfielder, WM = wide midfielder, WF = wide forward, CF = centre forward. All values have been rounded up or down to create integers. *P* < 0.05^a^; *P* < 0.01^b^

### Possession Status Profiles

Defensive positions such as CB and DM covered a greater proportion of their overall distance out-of-possession compared to more offensive positions such as CF (42–43% vs 38%; *P* < 0.05; ES: 0.4–0.5). Although, CF covered a higher proportion of their overall distance covered in-possession compared to CB, WD, DM, this just failed to reach statistical significance (40% vs 37–38%; *P* > 0.05; ES: 0.3–0.4). Defensive positions such as CB and DM covered a greater proportion of their distance at higher intensities (≥19.0 and ≥23.0 km · h^-1^) out-of-possession than offensive positions such as WM, AM, WF and CF (72–75% vs 40–48%; *P* < 0.01; ES: 1.9–2.4 and 75–82% vs 35–45%; *P* < 0.01; ES: 1.3–1.4, respectively). In contrast, WM, AM, WF and CF covered a higher proportion of their distance at higher intensities (≥19.0 and ≥23.0 km · h^-1^) in-possession compared to CB and DM (51–59% vs 20–26%; *P* < 0.01; ES: 1.1–2.8 and 54–62% vs 15–22%; *P* < 0.01; ES: 1.5–2.4, respectively).

### Half-by-Half Comparisons

Figures 3A-C highlight the half-by-half differences across position on a per minute basis. Figure 3A illustrates that most positions demonstrated a second half reduction in total distance covered compared to the first half (*P* < 0.01; ES: 0.6–1.0), with WF the only exception (*P* > 0.05; ES: 0.4). Figures 3B-C demonstrate that a decline between halves for high-intensity distance (≥19.0 km · h^-1^) was only evident for CF (*P* < 0.01; ES: 0.5), whilst a decline between halves for sprinting distance (≥23.0 km · h^-1^) was only found for WD (*P* < 0.05; ES: 0.3).

**FIG. 3A:**
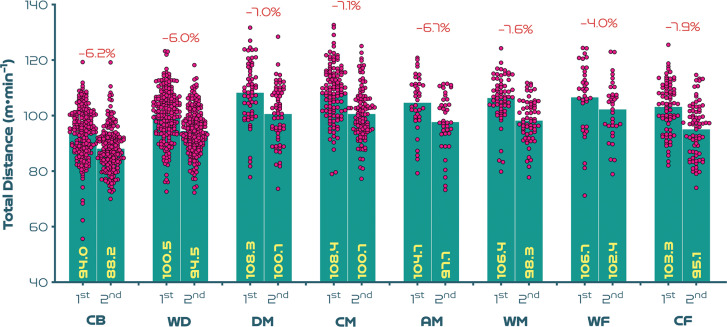
Positional half-by-half total distance per minute and percentage decrease between halves at the FIFA Women’s World Cup 2023. Data normalised to only include players who completed 90+ minutes (excludes goalkeepers and extra time). CB = centre back, WD = wide defender, DM = defensive midfielder, CM = central midfielder, AM = attacking midfielder, WM = wide midfielder, WF = wide forward, CF = centre forward. 1st = first half, 2nd = second half. Negative symbol = less distance in second half, Positive symbol = more distance in second half.

**FIG. 3B:**
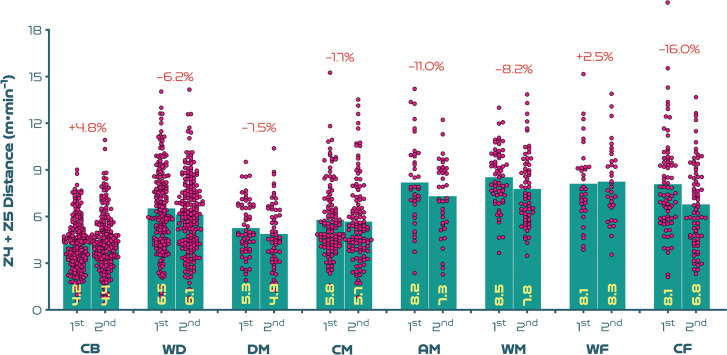
Positional half-by-half high-intensity distance (Zones 4 + 5 [Z4 + Z5], ≥19.0 km · h^-1^) per minute and percentage increase/decrease between halves at the FIFA Women’s World Cup 2023. Data normalised to only include players who completed 90+ minutes (excludes goalkeepers and extra time). CB = centre back, WD = wide defender, DM = defensive midfielder, CM = central midfielder, AM = attacking midfielder, WM = wide midfielder, WF = wide forward, CF = centre forward. 1st = first half, 2nd = second half. Negative symbol = less distance in second half, Positive symbol = more distance in second half.

**FIG. 3C:**
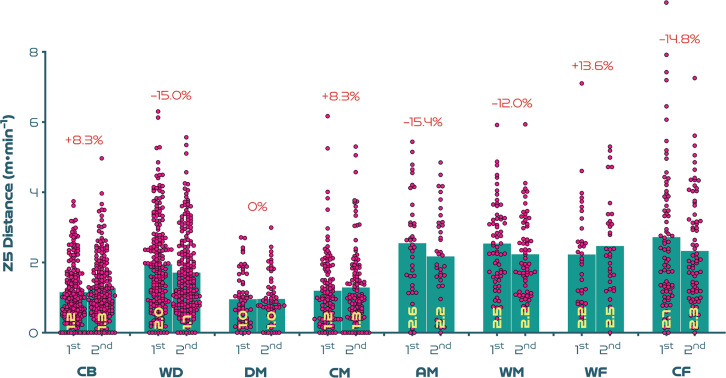
Positional half-by-half sprint distance (Zone 5 [Z5], ≥23.0 km · h^-1^) per minute and percentage increase/decrease between halves at the FIFA Women’s World Cup 2023. Data normalised to only include players who completed 90+ minutes (excludes goalkeepers and extra time). CB = centre back, WD = wide defender, DM = defensive midfielder, CM = central midfielder, AM = attacking midfielder, WM = wide midfielder, WF = wide forward, CF = centre forward. 1st = first half, 2nd = second half. Negative symbol = less distance in second half, Positive symbol = more distance in second half.

### A Within Tournament Sprinting Analysis Across Three FIFA Women’s World Cups

**FIG. 4:**
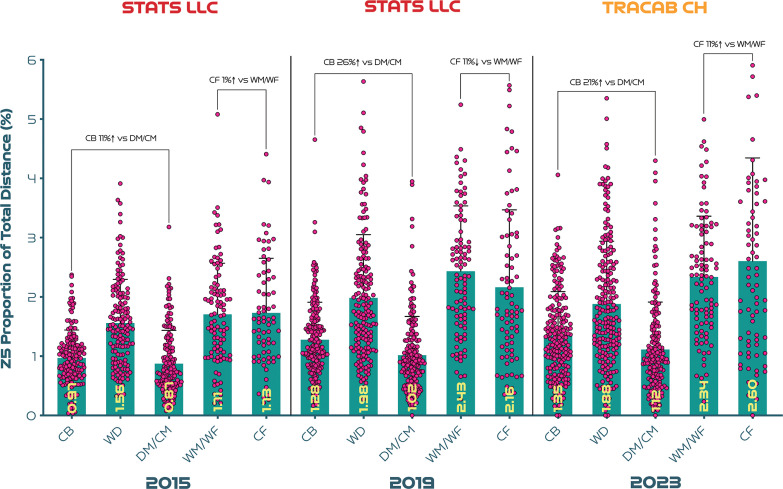
Sprinting distance (Zone 5 [Z5], ≥23.0 km · h^-1^) as a proportion of total distance across the last three editions of the FIFA Women’s World Cup. Numerous caveats are associated with comparing data from different optical tracking systems and, to account for these, only within- tournament trends have been presented. Some positions were combined as the 2015 and 2019 data sets did not spilt data in eight outfield roles. CB = centre back, WD = wide defender, DM/CM = defensive/central midfielder, WM/WF = wide midfielder/forward, CF = centre forward. Please note that attacking midfielders were not included in the analysis as they were not options in the 2015 and 2019 data sets, hence their omission.

Figure 4 highlights a clear trend within each competition as CB and DM/CM covered the lowest sprint distance as a proportion of total distance. Given the similarities across tournaments, one could gain longitudinal insights by comparing the magnitude of change across time for these roles. For instance, in Canada 2015, CB performed 11% more sprinting than DM/CM (*P* > 0.05; ES: 0.2). However, in France 2019 in addition to Australia and New Zealand 2023, CB performed 21–26% more sprinting than DM/CM (*P* < 0.01; ES: 0.3–0.4). Another obvious trend within each competition was that CF and WM/WF covered the most sprinting distance as a proportion of total distance. In Canada 2015, there was a negligible difference between these positions (ES: 0.0; *P* > 0.05). However, in France 2019, CF sprinted 11% less than WM/WF (ES: 0.2; *P* > 0.05) but in Australia and New Zealand 2023 this trend reversed as CF covered 11% more sprinting than WM/WF (ES: 0.2; *P* > 0.05). Although this later trend failed to reach statistical significance, it indicates a large relative shift (-11% to +11%).

## DISCUSSION

This study was the first to benchmark the position-specific match demands at the FIFA Women’s World Cup Australia and New Zealand 2023. This was accomplished through combining FIFA’s Enhanced Football Intelligence metrics alongside match physical performance measures. This added context to the physical trends so more nuanced positional insights could be determined. Moreover, highlighting pertinent examples of individuals in selected positions may also aid our understanding of the dynamic interplay between physical, technical and tactical factors.

Data demonstrated that central and defensive midfielders covered the greatest total distance and in contrast centre backs covered the lowest overall ground at the FIFA Women’s World Cup 2023. Similar findings were found at both Canada 2015 and France 2019 tournaments [2]. As differences were comparable across three Women’s World Cups (13–15% higher in central midfielder’s vs centre backs), it appears this trend remains stable at the highest standard of women’s football. Midfielders are renowned for their all-round work-rate [10], and to cite examples, Spain’s Teresa Abelleira and Zambia’s Ireen Lungu covered around 12.3–13.3 km during selected games. Despite contrasting possession profiles, these two players set the upper total distance requirement for contemporary international female midfielders. These benchmarks could be related to the midfield position being dynamic during all phases of play, particularly at the low to moderate speeds [2, 11–13, 24]. To support this assertion, FIFA’s Enhanced Football Intelligence metrics demonstrated that midfielders performed a high number of passes, offers to receive, especially in front of lines and applied pressures. In contrast, centre backs have a more sporadic activity profile, with more of their activity occurring out-of-possession [2], hence the lower total distance covered. Furthermore, centre backs are acutely influenced by the opposition’s attacking quality and work-rate [25]. For example, the Philippines’ Jessika Cowart covered around 11.6 km against Switzerland, which was the highest total distance for this position of the competition. In that specific match, Switzerland’s attacking dominance resulted in their highest figures for movements in behind and final-third phase counts of their tournament [21]. To nullify this, the Philippines’ defensive unit had to respond with their greatest number of applied pressures of their three games, which may explain Cowart’s physical output. On the contrary, Canadian centre back Kadeisha Buchanan covered only 7.8 km against Nigeria, which was the lowest total distance of the tournament. Buchanan may have been less active in that game as her team were consistently on the front foot, as evidenced by Canada registering their highest progression and final-third phase counts of their tournament [21]. The examples above clearly highlight the impact of numerous contextual factors on the demands of certain playing positions.

Researchers suggest that high-intensity running during matches is a valid measure of physical performance in women’s football because of its strong association with training status [18, 26] and is a distinguishing characteristic between different standards of player [10]. Moreover, others have demonstrated the instrumental role that intensity plays in the pre-ample to game-changing moments [27, 28]. Data revealed that wide midfielders and forwards covered around 40–90% more distance at high-intensity (≥19.0 km · h^-1^) than centre backs in addition to central and defensive midfielders. The average high-intensity game distance using fixed speed zones for both of these wide roles was around 0.8 km. However, at the upper level, Zambia’s Racheal Kundananji and Haiti’s Roseline Éloissaint covered around 1.4–1.6 km, underscoring how demanding these wide roles are in the modern women’s game. Although physical fitness is related to the high-intensity distances covered by elite female players during matches, this relationship remains complex [18]. Whereas superior intermittent endurance capacity has been found in female players operating in wide versus central roles [26], this work could now be considered outdated. Recent research has also demonstrated that the skeletal muscle phenotype of female players appears to be associated with their high-intensity match performances [29], thus wide players could be more likely to possess a certain phenotype profile. Although adopting such a reductionist perspective can be problematic in such a complex sport [15–17], as it is likely that this finding is multifaceted. For instance, the high-intensity nature of wide players could be related to the space afforded to them along the flanks, enabling them to accelerate to higher speeds when tactically required [11, 24]. Moreover, wide players may be more active at high-intensity due to modern tactics, with teams now commonly employing compact mid-blocks (e.g., narrow defensive shape in the mid third of the pitch) that prompt the opposition to use the flanks to create chances [11, 24, 30]. Whereas, central players operate in player compact pitch areas, which limits their ability to accelerate into space [24, 27]. Particularly if required to maintain tactical discipline and hold a position in central areas for extended periods [11]. This may have resulted in central roles performing lower accumulated high-intensity distances of between 0.4–0.6 km per match at the competition.

A novel finding of the present study was that centre forwards covered the most sprint distance (≥23.0 km · h^-1^) during matches. For instance, centre forwards sprinted an average of 263 m per game at the tournament, which was 7–8% more than their attacking counterparts. Data demonstrated that most of this sprinting was while their team was in-possession of the ball. This is unsurprising as a key role of a centre forward is to move rapidly to receive the ball in attacking areas, particularly making runs into the space in behind the opposition defence [12, 13, 17]. Accordingly, FIFA’s Enhanced Football Intelligence metrics indicated that centre forwards amassed around 45–80% more movements in behind than other attacking positions at this tournament. This was often coupled with defending intensely from the front via applied pressure. Both of these factors could go some way towards explaining the evolution observed in centre forwards’ sprinting outputs. Indeed, centre forwards covered a staggering 39–163% more sprinting than defensive positions at the FIFA Women’s World Cup 2023. This highlights the need for practitioners to develop drills that require players to sprint in a position-specific way [14].

The top ten speeds attained across position at the tournament revealed that some players attained speeds of >32.0 km · h^-1^, which agrees with data from European competitions [31]. There was a positional divide, with a higher proportion of these top speeds been produced by offensive as opposed to defensive players (70% vs 30%). Similarly, average top speeds attained in games were fastest for attacking midfielders, wide midfielders, wide forwards and centre-forwards, while defensive and central midfielders had the lowest top speeds. Slight discrepancies are evident between the top speeds attained during sprint testing and those reported in the present study [32]. Disparities would be expected given the different collection methods (tracking system vs timing gates) and that top speeds attained during testing don’t necessarily agree with those attained during matchplay [33]. Moreover, caution is needed when interpreting top speeds during match-play due to the limited sampling frequency of the optical tracking system used. Despite such complexities, these findings could attest to the importance of speed development across all positions in women’s football, especially for attacking players.

Practitioners can use match physical performance trends as a blue-print to create position-specific drills [14, 17]. Although complex, these drills should tax the relevant physical attributes, while simultaneously mimicking commonly occurring position-specific technical and tactical actions [13]. Over time this approach may develop specific adaptations in players that enables them to better fulfil their duties across the whole game (volume) and particularly during intensified match-play periods (intensity) [34]. To facilitate this process for women’s football, it could be advantageous to correlate distinct physical dimensions (volume vs intensity) to determining specific positional characteristics. Using such an approach indicated most centre backs exhibited low volume and intensity characteristics, which agreed with observations from the FIFA Men’s World Cup 2022 [11]. Wide defenders typically covered a low volume but intensity varied somewhat. As offensive wing-backs as opposed to traditional full-backs are usually higher on the intensity continuum due to more overlapping, support play, dribbling etc [12], this could account for some of this variation. Central and defensive midfielder’s physical performances emulated one another, implying a prerequisite of both was to cover considerable volume during matches. Although intensity varied more in central midfielders due to box-to-box and more traditional midfielders being included in the same positional category [12]. Wide midfielders, wide forwards and attacking midfielders exhibited both high volume and intensity qualities but substantial variation existed for these roles. Finally, centre forwards were the position that fluctuated the most for volume and intensity. This may indicate the existence of various centre forward archetypes within the sample (e.g., false 9s, supporting forwards, target forwards, out-and-out strikers, etc). Although a practitioner could extrapolate from such trends, the degree of physical preparation needed, caution is warranted given the extensive variation across most positions. This is especially evident for centre forwards, as players resided fairly equally across quadrants. Given such a data spread, conditioning practices should always be in alignment with the age and capabilities of the players in these roles, in addition to the playing style adopted by each team [11].

Elite female players have been found to cover less distance both in total and at higher intensities in the second compared to the first half of matches [2, 4, 18]. Previously, trends were based on a reasonably similar duration played in the two halves. However, new directives to added time in the FIFA Women’s World Cup 2023 resulted in much longer second halves. If the intense nature of women’s football was played over longer durations, then some degree of fatigue might be expected towards the end of games, especially for the most demanding positions [35]. Accordingly, second-half declines in high-intensity and sprint distances on a per minute basis were more marked for attacking midfielders and centre forwards. While centre backs, defensive midfielders, central midfielders and wide forwards illustrated less pronounced second-half declines or, in some cases, maintained or even increased their second-half intensity. As high-intensity actions during elite female games have been found to heavily deplete muscle glycogen stores in Type II fibres [35], these positional trends seem logical. Thus, limited energy availability in the second half could have resulted in more pronounced performance declines for those sprinting more overall in tournament games (e.g., centre forwards and attacking midfielders). This may also partly explain why players in positions completing less sprinting across games were able to maintain or even increase their second-half sprint performances (e.g., centre backs and central midfielders). Wide forwards were the only position to deviate from this trend. Due to the demanding nature of this role, it could be assumed that wingers possess superior physical capabilities compared with other positions [26]. However, this finding could also be related to wide forwards being more active during the second half of games, as the onus to create attacking opportunities increases, particularly via crosses [11, 19, 30].

The evolution of match physical performances has been quantified in various competitions [24, 36]. Specifically in the women’s game, the amount of sprinting increased by around 20–50% across various positions between the Canada 2015 and France 2019 FIFA editions [2]. Thus, it is crucial to map such advancements, especially given the growth of the women’s game in recent years. However, employing different optical tracking systems across major international tournaments can hinder the ability to survey such performance developments [37]. The present study analysed the largest sample of international female players published to date across three FIFA Women’s World Cup editions. Despite identical speed zone across competitions, the optical tracking systems differed and unfortunately no between tournament positional comparisons could be made. Alternatively, a within tournament positional comparison revealed that centre backs and centre forwards demonstrated more pronounced changes in their relative sprint distance compared to other positions in Australia and New Zealand 2023 and France 2019 compared to Canada 2015. Accordingly, the sprint profiles of these two positional adversaries could be inextricably connected (e.g., centre backs vs centre forwards). As centre forwards increasingly attack space or run with the ball, it follows that centre backs are required to defensively react through various runs to press, cover or track back [11–13, 17]. As the present study found diametrically opposed sprinting profiles in- and out-of-possession for these two roles, this could further support that assertion. Although the author has been overly cautious in this analysis by only observing within-competition positional trends for relative measures, the reader should still be cognisant of the numerous caveats associated with comparing trends from different technologies and view the trends with some caution.

To provide a balanced perspective, the major shortcomings of the present work should be examined. Firstly, it is worth noting that the data provider assigned the specialised positional roles. These were based on the tactical systems adopted at the start of games and the same players may have been assigned different roles across various matches. Although the author agreed with most role assignments by the data provider some disagreement was evident at times. Secondly, the physical data was limited to locomotor metrics and thus omits crucial information on position-specific acceleration and change of direction profiles [38]. Thirdly, the present study used both FIFA’s Enhanced Football Intelligence metrics and pertinent individual examples to add much-needed context. However, to provide more nuanced and actionable insights, this analysis preferably needed the physical data to be directly synchronised with various tactical phases and/or scenarios [15]. Fourthly, additional contextual factors (e.g., score, match importance, level of opponent and weather conditions) should have been explored to further understand the complexity associated with the demands of the game. Finally, although the optical tracking system used in the present study has been found to be valid [22], more research is needed to verify its reproducibility. In line with this, the reliability and validity of FIFA’s Enhanced Football Intelligence metrics should also be quantified in future studies.

## CONCLUSIONS

This study demonstrated the marked differences in match demands across specialised positions at the FIFA Women’s World Cup 2023.

These trends could be used to benchmark international female players and to form a basic blue-print for training requirements that replicate the most important facets of performance across specific roles.
